# Prevalence and Characterization of *Staphylococcus aureus* in Growing Pigs in the USA

**DOI:** 10.1371/journal.pone.0143670

**Published:** 2015-11-24

**Authors:** Jisun Sun, My Yang, Srinand Sreevatsan, Peter R. Davies

**Affiliations:** Department of Veterinary Population Medicine, College of Veterinary Medicine, University of Minnesota, St. Paul, Minnesota, United States of America; Animal Health and Veterinary Laboratories Agency, UNITED KINGDOM

## Abstract

A decade of research of methicillin-resistant *S*. *aureus* (MRSA) in pigs shows that the prevalence and predominant genotypes (i.e., ST398, ST9, ST5) of MRSA vary widely geographically, yet knowledge of the epidemiology of *S*. *aureus* generally in swine remains rudimentary. To characterize *S*. *aureus*, including MRSA, in the US swine industry, we sampled 38 swine herds in 11 states in major swine producing regions. The herds sampled included pigs sourced from 9 different breeding stock companies, and the sample was likely biased towards larger herds that use regular veterinary services. Twenty nasal swabs were collected from 36 groups of growing pigs by 36 swine veterinarians, 2 more herds were sampled opportunistically, and a historically MRSA-positive herd was included as a positive control. *S*. *aureus* was detected on 37 of the 38 herds, and in 77% of pigs sampled. Other than the positive control herd, no MRSA were detected in the study sample, yielding a 95% upper confidence limit of 9.3% for MRSA herd prevalence. All but two (ST1-t127; ST2007-t8314) of 1200 isolates belonged to three MLST lineages (ST9, ST398, and ST5) that have been prominent in studies of MRSA in pigs globally. A total of 35 spa types were detected, with the most prevalent being t337 (ST9), t034 (ST398), and t002 (ST5). A purposively diverse subset of 128 isolates was uniformly negative on PCR testing for major enterotoxin genes. The findings support previous studies suggesting a relatively low herd prevalence of MRSA in the US swine industry, but confirm that methicillin susceptible variants of the most common MRSA genotypes found in swine globally are endemic in the US. The absence of enterotoxin genes suggests that the source of toxigenic *S*. *aureus* capable of causing foodborne enterotoxicosis from pork products is most likely post-harvest contamination.

## Introduction

Prior to the recognition that pigs and other livestock species can be reservoirs of methicillin resistant *Staphylococcus aureus* (MRSA) [1], *S*. *aureus* was considered a relatively unimportant organism in swine. Mounting concerns regarding the occupational and public health implications of MRSA in livestock populations have stimulated research of MRSA in animals, and particularly pigs, in many countries[2–6]. Although the ST398 lineage of *S*. *aureus* was the first designated to be ‘livestock associated’ in Europe, broader investigations have confirmed that MRSA of other MLST types (e.g., ST9, ST5) also occur in swine populations[7–9]. Furthermore, the relative prevalence of these lineages, and subtypes within lineages, appears to vary geographically[10,11]. ST398 variants have been predominant in studies of pigs in Europe, ST9 in studies from most Asian countries, while both ST398 and ST5 have been relatively common in North American studies[8,12–15]. Within the ST398 lineage in Europe, spa types t108 and t011 have been predominant in the Netherlands, while spa type t034 is predominant in Denmark[11,16,17]. Similarly, the predominant spa types of ST9 MRSA isolated from pigs vary among Asian countries[18].

Until recently, research of *S*. *aureus* in pigs has been heavily focused on MRSA, with relatively little attention given to the ancestral organism[19,20]. The need for a more holistic approach to *S*. *aureus* epidemiology is illustrated by a recent study in China in which all MRSA isolates from pigs were ST9, but 61% of methicillin sensitive *S*. *aureus* (MSSA) isolates from the same population were ST398[21]. There has been no comprehensive study to determine the prevalence of MRSA in pigs in the USA or Canada, but several studies have reported observations on MRSA and/or MSSA in geographically limited studies using convenience sampling (Table 1).

**Table 1:** Overview of *Staphylococcus aureus* prevalence in the USA.

Type	Year	Pig Source (age)	Location	# of herds sampled		Herd Prevalence	Animal Prevalence	Spa types	MLST	Ref
Commercial	2009	9, 12, 15, 18, 21, and 24 weeks	IA, IL	2	**MRSA**	50% (1/2)	49% (147/299)	NA	ST398	[13]
	2011	Small, Medium, Large size of pigs on USDA standard	CT	35	**MRSA**	14% (5/35)	3% (8/259)	t008, t007, t011	ST8, ST398	[20]
					MSSA	43% (15/35)	30% (85/259)	t337, t034, t334, t4529 t8760, t1166	NR	
	2012	Various ages	IA	40	**MRSA**	30% (12/40)	18% (34/194)	t002, t034, t548	ST398, ST5	[8]
	2012	Market age	OH	10	**MRSA**	50% (5/10)	3% (7/240)	t034, t337	ST398, ST9	[15]
	2013	6 and 18 weeks	IL(+) IA(+) MN, NC, OH	45	**MRSA**	9% (4/45)	5% (50/1085)	t034, t002, t337, t571 t3446, t002	ST398, ST5 ST9	[12]
	2015	From farrowing to Finish	MN	2	MSSA	100% (2/2)	91.1% (175/192)	t034, t337, t7331, t2462, t3446, t001, t571, t1255, t526	ST398, ST5, ST9	[23]
	**Total**			134	**MRSA**	20% (27/134)	9% (246/2269)			
Others	2011	Backyard-raised pigs	MI	50	**MRSA**	2% (1/50)	4% (2/53)	NA	ST5	[30]
	2012	Statefairs A and B	MN, IA	N/A	**MRSA**		2% (2/103, Fair B)	t3075, t337	ST398 ST2136	[29]

MRSA in pigs in North America was first reported in 9 of 20 swine herds in Ontario, Canada[14], where ST398 related spa types comprised 75% of isolates, but 3 closely related spa types (t002, t067, t653) likely to belong to the ST5 lineage were also found in 3 herds. Subsequently, a more representative study across Canada found MRSA in fewer herds (5 of 46; 11%), again with ST398 variants predominating and ST5 variants accounting for most of the remaining isolates[22]. In the USA, MRSA in pigs (ST398) was first reported in one of 2 production systems in the US Midwest[13]. A broader study of 45 herds (including 21 herds classified as ‘antibiotic free’) in 4 US states identified 4 (9%) MRSA positive herds (ST398, ST5 and ST9), [12] although 2 herds were affiliated with the one system previously known to be positive from an earlier study [13]. Frana et al (2013) detected MRSA in 12 (30%) of 40 herds in Iowa, with ST5 isolates (t002, t548) comprising the majority (82%) detected in pigs, and ST398 the remainder[8]. Similarly, a study of 10 herds in Ohio found that 3 (30%) herds were MRSA positive, and detected the ST5, ST398, and ST9 lineages[15]. Notably, MRSA prevalence in pigs was low overall (2.9%) in that study, even among pigs from positive herds (7 of 72 pigs; 10%), in contrast with most reports in which pig prevalence typically exceeds 50% on positive herds[8,12,14]. Thus, observed prevalence and diversity of MRSA in US pig herds have varied among studies, and methicillin susceptible *Staphylococcus aureus* (MSSA) isolates have not been well characterized other than one study of small scale producers in Connecticut[20].

To guide the design of the current study, we conducted a pilot investigation of *S*. *aureus* ecology in two multiple-site production systems in Minnesota[23]. Neither herd yielded MRSA isolates, but *S*. *aureus* was detected in 91% of pigs sampled, with all MSSA isolates belonging to the ST398, ST9, or ST5 subtypes. Within systems, multiple spa types (>5 types) and MLST types (2 or 3 sequence types) occurred, and individual pigs frequently carried multiple *S*. *aureus* variants. The goal of the current study was to estimate the prevalence and diversity of *S*. *aureus* in growing pigs in a geographically diverse sample of commercial herds in the USA. Given that pork products, particularly ham, are often implicated in cases of staphylococcal enterotoxicosis in people[24], we also tested for major enterotoxin genes A to E to assess the potential importance of the swine reservoir as a source of enterotoxigenic *S*. *aureus*.

## Materials and Methods

### Selection of Herds and Animals

A cross sectional study was conducted on 36 swine herds located in 11 states of the USA (Midwest: IA, IL, IN, MI, MN, NE, SD and Non-Midwest: AL, NC, PA, TX). All procedures were approved by Institutional Animal Care and Use Committee (IACUC) at University of Minnesota (1303-30452A), and sampling was conducted from June 2013 to November 2014. Herds were selected by 36 swine veterinarians, who were purposively chosen from a cohort of swine veterinarians participating in a separate longitudinal study of MRSA colonization and infection in people[25]. To maximize diversity in the herds included, each veterinarian selected by convenience one client herd for sampling, and no more than 2 herds were serviced by the same veterinary clinic. The veterinarians were mailed sampling instructions for obtaining nasal swabs from 20 growing pigs aged 4 weeks or older in one client herd. Nasal swabs were mailed to University of Minnesota for processing. The same process was used to collect samples from one positive control herd known to be MRSA positive from previous studies[12,13]. Nasal swab sampling was also conducted opportunistically on pigs at another 2 herds in Minnesota visited for educational purposes by our group.

The sample size of 20 pigs per herd was calculated based on the pilot study that found the apparent prevalence of *S*. *aureus* in nasal swabs of pigs to be greater than 60%[23]. Based on an expected prevalence of 60%, it was anticipated there would be an average of 12 positive isolates per herd sampled, and that at least 8 isolates would be obtained in 97% of herds sampled. A total of 739 pig nasal swabs were collected on the 36 veterinary selected herds (20 pigs on 29 of the herds; 21 on 1 herd; 22 on 4 herds; and 25 on 2 herds). Twenty pigs were also sampled on the positive control herd, and on the two educational visits swabs were collected from 37 and 30 pigs respectively. For detection of MRSA at the herd level, a conservative estimate of within-herd prevalence of 10% of MRSA positive pigs would yield and herd sensitivity of 87.8% if 20 pigs were tested. In turn, sampling of 36 herds yields 97% probability of detecting at least one infected herd if 10% of herds were positive.

### Bacterial Isolation and Characterization

Isolation of *S*. *aureus* was performed using the methods described previously[23]. Nasal swabs were double enriched in Mueller-Hinton broth (BBL^™^, MD, USA) supplemented with NaCl (6.5%) and in Phenol-Red Mannitol broth (BBL^™^, MD, USA) supplemented with 4ug/ml Oxacillin (Sigma-Aldrich, MO, USA). Broths showing a color change to yellow were selected for inoculation on chromogenic agar plate (BBL CHROM agar MRSA, MD, USA) and Factor plate (Veterinary Diagnostic Laboratory, University of Minnesota, MN, USA) to culture MRSA and *S*. *aureus*, respectively. Two colonies per sample were collected for further characterization. DNA was extracted from one colony on the plate with 19.5μl 10mM Tris-HCl and 0.5μl Lysostaphin (both Sigma-Aldrich, MO, USA) at 37°C for 30 min. PCR were used to detect the *mecA* gene and perform *spa* typing. The primers for the *mecA* gene were [F: 5’ GTA GAA ATG ACT GAA CGT CCG ATA A 3’, R: 5’ CCA ATT CCA CAT TGT TCG GTC TAA 3’], and the *spa* gene [F: 5’ AGA CGA TCC TTC GGT GAG C 3’, R: 5’ GCT TTT GCA ATG TCA TTT ACT G 3’]. PCR master mix (USB HotStart-IT FideliTaq, affymetrix, CA, USA) was used to amplify DNA under the following condition: 95°C for 2min, 94°C for 30s, 55°C for 30s, 72°C for 1min with 30 cycles and 72°C for 10 min[26]. All PCR products were visualized on 1% agarose gel with SYBR Safe dye in 1X TAE buffer (Tris-Acetate-EDTA, Thermo Fisher Scientific Inc., MA USA) for 40min at 200 V.

### Molecular Typing and Analysis

All selected *S*. *aureus* isolated were subtyped using *spa* typing [26]. After amplification of the *spa* gene, PCR products were cleaned up with Illustra Exoprostar, (GE Healthcare Bio-sciences, PA, USA) then submitted to the Biomedical Genomic Center (BMGC, University of Minnesota, MN, USA) to obtain gene sequences. After aligning sequences using Sequencher 5.1 software (Gene Codes Corporation, MI, USA), each sequence was submitted to Ridom spa typing database (http://spa.ridom.de/index.shtml).

Multi-locus sequence typing (MLST) of *S*. *aureus* was performed following the methods previously reported [27]. Briefly, seven housekeeping genes (carbamate kinase (arcC), shikimate dehydrogenase (aroE), glycerol kinase (glpF), guanylate kinase (gmk), phosphate acetyltransferrase (pta), triose-phosphate isomerase (tpi), and acetyl coenzyme A acetyltransferase (yqiL)) were amplified and sequenced. Specific allelic numbers of each isolate and sequence type were obtained via the MLST database of *S*. *aureus* (http://saureus.mlst.net).

### Detection of Enterotoxin Genes

The presence of enterotoxin genes A to E was tested by PCR in a subset of 128 isolates purposively selected to include at least one isolate of each spa type detected on each herd (educational herd samples excluded). A multiplex PCR was used to detect genes for *S*. *aureus* enterotoxins A (*sea*), B (*seb*), C (*sec*), D (*sed*), and E (*see*). Primer sequences for the five enterotoxin genes were selected using published research[28]. The primer mix to run a single PCR reaction contained 5.5μL nuclease-free water, 12.5μL HotStart-It Fideli Taq Master Mix, (Affymetrix), 0.5μL of each 10μM forward and reverse enterotoxin primers (for *sea*, *seb*, *sec*, and *see*), 1.0μL of 10μM *sed* primers, and 1.0μL of extracted *S*. *aureus* DNA. DNA amplification was conducted with the following thermal cycling profile: an initial denaturation at 94°C for 2 min was followed by 30 cycles of amplification (denaturation at 94°C for 2 min, annealing at 56°C for 2 min, and extension at 72°C for 1 min), ending with a final extension at 72°C for 5 min. PCR products were visualized under the same conditions used for *mecA* PCR. *S*. *aureus* isolates ATCC 13565, ATCC 14458, ATCC 19095, ATCC 23235 and ATCC 27664 were used as positive controls for enterotoxin genes A to E, respectively.

### Survey Questions and Statistical Analysis

Each swine veterinarian was requested to complete an online questionnaire to obtain information about the herd from which samples were collected. The questionnaire was administered via Survey Monkey (http://www.surveymonkey.com) and questions included herd size, age of pigs sampled, type of herd, genetic origin, and number of sources providing pigs to the group sampled (surveys were not conducted on the 2 educational visits). Descriptive statistics of the detection of *S*. *aureus* by herd characteristic were calculated as prevalence at the pig level, using prevalence ratios to compare subgroups. Mixed models to account for clustering at herd level could not be conducted due to sparsity of data in some categories, resulting in quasi complete separation. Within herd prevalence was highly skewed, therefore univariable analyses of associations between within-herd prevalence of *S*. *aureus* and herd characteristics were conducted by Kruskal-Wallis one-way analysis of variance using Statistix 10.0 (Analytical Software, Tallahassee, FL, USA).

## Results

**Fig 1:**
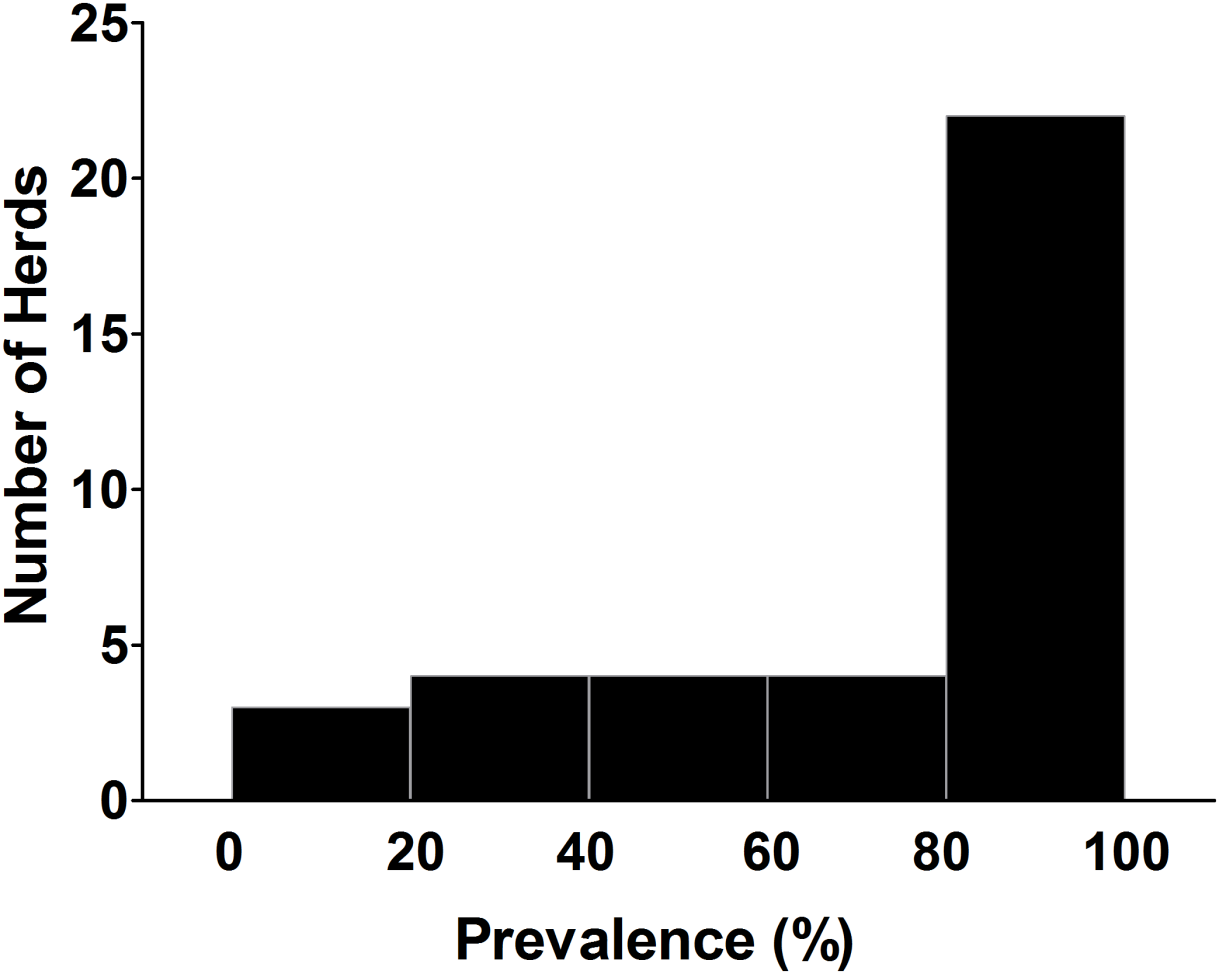
Histogram of prevalence of *Staphylococcus aureus* in 38 herds.

Thirty-six herds from 11 states in the USA were sampled by veterinarians, mostly (29 of 36, 81%) in the Midwest region where pig production is concentrated, plus 2 additional herds in Minnesota. Completed surveys were obtained for 35 of the 36 herds (97%), and for the MRSA positive control herd. Excluding 7 herds where the genetic sources of pigs were unknown, pigs on the herds sampled by veterinarians originated from the following breeding stock suppliers: Pig Improvement Company (10 herds); Choice Genetics (5 herds); Danbred (4 herds); Genetiporc (3 herds), Fast Genetics, Genesus Genetics, and Topigs/Norsvin (2 herds each); Smithfield Premium Genetics and Hypor (1 herd each). Herd sizes ranged from 40 to 12,000 head and most (22 of 36, 61%) were nursery herds. Mixing of pigs from multiple sources was not widely practiced, with 29 herds (81%) receiving pigs from a single source. The age of pigs sampled ranged from 4 to 20 weeks. Overall among the veterinary sampled herds, 739 nasal swabs were collected, of which 558 (76%) were culture positive for *S*. *aureus*, and positive pigs for *S*. *aureus* were detected on 35 of the 36 herds (97%). However, no MRSA were detected in any of the herds sampled, apart from the positive control herd on which all 20 pigs were positive for MRSA. The prevalence of *S*. *aureus* at herd level varied from 0 to 100% (Fig 1). For the majority of herds (60%, 21/35) prevalence exceeded 80%, including 12 (34%) herds with all pigs positive, while prevalence was less than 50% on only 7 herds. The two additional herds visited were also negative for MRSA, and had high prevalences (90%, 100%) of *S*. *aureus*.

### Molecular Characterization of *S*. *aureus*

Among the 35 *S*. *aureus* positive herds sampled by veterinarians, and the 2 opportunistically sampled herds, there was considerable diversity found with 35 spa types detected across 5 MLST sequence types (Table 2; S1 Table). The most predominant spa types (sequence type) were t337 (ST9), t034 (ST398) and t002 (ST5) which together accounted for 54% (653 of 1200) of all isolates. Seven spa types (t3232, t2582, t5883, t1793, t5462, and 2 unknown types) that were detected only once were closely related to the predominant spa type in their respective herd. For example, t2582 (ST398) was found on a herd where t034 was the predominant type. The repeat succession in the *spa* genes of t034 and t2582 comprised X1-K1-A1-O1-A1-O1-B1-Q1-O1 and X1-K1-A1-O1-A1-O1**-A1-O1-**B1-Q1-O1 respectively, suggesting that an insertion-deletion event of an A1-O1 repeat likely occurred in the population. All MRSA isolates in the positive control herd were t034 (ST398), as reported previously [12,13].

**Table 2:** MLST and spa types of *S*. *aureus* (n = 1200) isolated from pigs in 38 herds.

MLST	Spa type	Number of isolates (%)	Number of herds
**ST9**	t337	384 (32)	**25**
	t3446	73 (6)	5
	t2498	59 (5)	3
	t2315	23 (2)	1
	t1334	14 (1)	1
	t2462	3 (0.3)	2
	unknown 1*	2 (0.2)	1
	t10494	2 (0.2)	1
	unknown 2*	1 (0.1)	1
	unknown 3*	1 (0.1)	1
	t3232	1 (0.1)	1
**ST398**	t034	152 (13)	**12**
	t571	138 (12)	4
	t1255	34 (3)	1
	t899	30 (3)	2
	unknown 4*	27 (3)	1
	t5838	19 (2)	1
	t14581	16 (1)	1
	unknown 5*	12 (1)	1
	t1419	12 (1)	1
	t011	10 (1)	3
	t11374	6 (0.5)	1
	t11241	4 (0.3)	1
	t2582	3 (0.3)	2
	t11744	2 (0.2)	1
	t2582	1 (0.1)	1
	t1793	1 (0.1)	1
	t5883	1 (0.1)	1
	t5462	1 (0.1)	1
**ST2007**	t8314	5 (0.4)	1
**ST1**	t127	7 (0.6)	1
**ST5**	t002	117 (11)	**9**
	t570	22 (2)	3
	t242	13 (1)	1
	t306	4 (0.4)	1

*Repeat succession of unknown types: Unknown1 (r07r16r23r23r02r12r17r23r02r34), Unknown2 (r07r16r16r16r23r23r02r12r23r02r34), Unknown3 (r07r16r16r23r02r12r23r02r34), Unknown4 (r08r475r2r25r2r25r34r34r25), unknown5 (r07r16r23r23r02r23r02r34).

### Associations between Herd Attributes and *S*. *aureus* Nasal Colonization in Pigs

Due to the absence of MRSA in all pigs tested, no analysis of MRSA occurrence was possible and analyses were limited to *S*. *aureus* prevalence. Also, as only one herd was negative for *S*. *aureus*, no analysis was possible for herd status (positive/negative). Based on prevalence ratios at pig level, unadjusted for clustering by herd, there was no indication of a difference in *S*. *aureus* prevalence by geographic region or herd size (Table 3). However, the data indicated that prevalence was lowest in nursery pigs compared to other farm types and in the youngest pigs 4–6 weeks old (noting that pig age is confounded with farm type, as nursery facilities only house young pigs), and there was some suggestion that prevalence was higher in commingled groups. Based on non-parametric analysis of variance of prevalence at herd level, prevalence was significantly lower in nursery facilities (P = 0.02), and tended to be positively associated with age (P = 0.07), but the effect of commingling was not significant (P = 0.82).

**Table 3:** Prevalence of *Staphylococcus aureus* in pigs by herd (n = 36) characteristics.

	% Herds (n)	Median herd Prevalence %	Prevalence Pig % (n)	Prevalence ratio Pig (95% CI)*
**Herd size**				
≤3,000	47 (17)	85	74 (253/344)	Ref
>3,000	47 (17)	95	77 (273/355)	1.05 (0.96–1.14)
Missing	6 (2)	.	.	.
**Type of farm**				
Nursery	61 (22/36)	60	66 (299/452)	Ref
Finishing	17 (6/36)	90	98 (120/122)	1.49 (1.39–1.59)
Wean to Finish	14 (5/36)	95	78 (82/105)	1.181 (1.05–1.33)
Farrow to Finish	8 (3/36)	100	95 (57/60)	1.44 (1.32–1.57)
**Pig source**				
Single	81 (29/36)	90	74 (439/595)	Ref
Comingle (2 sources)	11 (4/36)	90	79 (63/80)	1.07 (0.94–1.21)
Comingle (≥ 2 sources)	8 (3/36)	95	88 (56/64)	1.19 (1.07–1.32)
**Geographic**				
Midwest ^†^	81 (29/36)	90	75 (446/597)	Ref
Non-Midwest ^‡^	19 (7/36)	100	79 (112/142)	1.056 (0.958–1.163)
**Sampling age (Weeks)**				
4–5	42 (15/36)	60	61 (186/305)	Ref
6–8	25 (9/36)	90	79 (147/185)	1.73 (1.52–1.97)
9–12	19 (7/36)	95	85 (125/147)	1.85 (1.63–2.10)
>12	14 (5/36)	100	98 (100/102)	2.14 (1.91–2.38)

* Pooled data not adjusted for clustering by herd

^†^Midwest: IA, IL, IN, MI, MN, NE, SD

^‡^Non-midwest: AL, NC, PA, TX

### Genotypic Diversity of *S*. *aureus* within Herds and Presence of Enterotoxin Genes

In 9 herds, all *S*. *aureus* isolates were of a single spa type, including six herds with ST9 variants [spa type t337 (5 herds) or t2498 (1 herd)], and 3 with ST398 variants [spa types t034 (2 herds); t5838 (1 herd)]. The most frequent scenario (15/37, 41%) was detection of two spa types in a herd, but 8 spa types were isolated from one herd. Genotypic diversity within herds did not appear to be related to genetic source, geographic location, herd size or age at sampling. All 128 isolates tested negative on PCR for enterotoxin genes A-E.

## Discussion

Despite over a decade of angst about MRSA associated with swine, the epidemiology of *S*. *aureus* generally in pigs has been remarkably neglected[23]. Furthermore, considerable uncertainty remains regarding MRSA prevalence in pigs in the USA where previous studies have had limited geographic scope and delivered mixed results (Table 1). The current study also cannot claim to be representative of the US swine population as herd selection was by convenience rather than formal random sampling from an identified national register of herds. However, the study has some features making it more likely to be representative of mainstream commercial swine production than any preceding studies, some of which purposively targeted small herds or niche populations more than conventional commercial herds[12,20,29,30]. Firstly, the current study was more geographically diverse, encompassing herds in 11 states (including the 6 leading pig producing states), mostly located in the major swine producing regions of the Midwest and South East. Secondly, by restricting sampling to one herd per veterinarian (and 2 herds per veterinary practice), we should have minimized local clustering of sampling within states which occurred in some earlier studies[8,15]. Using veterinarians to select herds would be expected to bias sampling towards larger herds which supply the bulk of the US pork supply, as larger herds are more likely to use veterinary services regularly. This is likely reflected in the fact that 9 of the study herds had more than 5000 animals while only 8 herds had less than 1000 animals. However, theoretical selection biases that are inherent in convenience sampling, such as the processes influencing the veterinarians to select a herd or the farmers being willing to participate, cannot be ruled out.

Perhaps more importantly, the current study included animals of diverse genetic provenance sourced from at least 9 different breeding stock companies, including most of the major genetic suppliers to the commercial industry. The pyramidal distribution of breeding stock in the swine industry is arguably a point of vulnerability that could lead to rapid dissemination of emerging pathogens throughout the industry, particularly for agents that typically do not cause disease in pigs[31]. However, our observations suggest that MRSA are not widely disseminated in major breeding stock populations in the USA at present. Furthermore, we found no clear association of MLST lineages of *S*. *aureus* with genetic suppliers, as multiple lineages were found among all genetic suppliers with more than one herd studied, and multiple MLST lineages were present in many herds.

An unexpected finding of the study was that no MRSA isolates could be detected in any of the 38 herds sampled. Based on zero positives among 38 herds in the study sample, the upper 95% confidence limit for herd prevalence, using exact binomial confidence intervals, is 9.25%. Higher herd prevalences (25 to 30%) have been reported in geographically restricted studies in the USA and Canada[8,14,15]. However, larger and more geographically representative studies in both these countries reported herd prevalence of the order of 10%[12,22]. Recent studies of occupationally exposed people in the USA also indirectly point to the likelihood that MRSA prevalence may be substantially lower in the US swine industry than in many European countries. In two small studies in North Carolina, MRSA prevalence was 7% in workers in both intensive livestock operations (3/41) and antibiotic free operations (3/42) [32], and one of 22 (4.5%) workers was positive in a longitudinal study[33]. A large study in Iowa found only 4 of 163 (2.5%) participants with current pig contact to be MRSA positive, which did not differ from prevalence (2.8%; 26 of 939) in participants without pig contact[34]. These prevalences of MRSA in US swine workers were only marginally higher than the 1.5% estimated for the general US population[35], but are an order of magnitude below MRSA prevalences reported in swine workers in community based studies in pig dense regions of Germany and the Netherlands [2,36,37]. Similarly, longitudinal studies in the Netherlands and USA indicate MRSA is much less prevalent in swine veterinarians in the USA (9%) than the Netherlands (44%), although both groups had comparable high prevalences (64%, 72%) of *S*. *aureus* in nasal swabs [25,38]. More definitive estimation of the herd prevalence of MRSA in the USA will require formal random sampling of the commercial swine industry. If such studies were to be pursued, based on current information we suggest they be designed with an expected prevalence of no more than 15%, and with the realization that positive herds may be clustered geographically[8].

Globally, the predominant lineages found in studies of MRSA in swine populations have been ST398 (most European and some North American studies), ST9 (most Asian studies) and ST5 (some North American studies). The current findings confirm that ST9, ST398, and ST5 MSSA are widely distributed in the US commercial swine population. Although diversity of spa types was evident within these lineages, each lineage was dominated by one spa type (ST9-t337; ST398-t034; ST5-t002) and these collectively comprised 54% of all *S*. *aureus* isolated. Perhaps unsurprisingly, t034 and t002 have been the predominant spa types among ST398 and ST5 MRSA found in pigs in North America, and 2 ST9-t337 MRSA isolates have also been reported[8,12,14,15]. Furthermore, ST9-t337 and ST398-t034 also constituted the majority of *S*. *aureus* isolates from 50 small pig farms in Connecticut[20], and from pigs sampled at agricultural fairs in two Midwestern states[29], suggesting a general predominance across multiple segments of the domesticated swine population. The complete absence of enterotoxin genes A-E among the isolates tested is consistent with another recent investigation of *S*. *aureus* in pigs [39] and suggests foodborne enterotoxicosis with these toxin types associated with consumption of pork products is most likely to result from post-harvest contamination with toxigenic *S*. *aureus*.

It is well established that particular lineages of *S*. *aureus* are more adapted to certain host species, and early observers of staphylococcal diversity among animal species posited that the host environment is probably the selective factor which determines the biological properties in host adapted staphylococci [40–42]. Although 3 MLST lineages accounted for almost all isolates in the original 36 study herds, it is likely that we underestimated diversity both within and among herds by sampling only one anatomical site in just 20 pigs per herd[23]. To date, the relatively common occurrence of ST5 MRSA and MSSA isolates in the swine reservoir appears to be unique to North America[8,12,14,15], although there are several reports of ST5 MRSA or MSSA from pigs in other countries [11,43,44]. Unlike the ST398 and ST9 lineages, which to date have played a negligible role in MRSA epidemiology of humans in the USA, the ST5 lineage has been a major component of hospital and community associated MRSA and MSSA infections worldwide [45]. While often deemed a ‘human adapted’ lineage in some studies of swine, an international study of 48 isolates from broilers in 8 countries found a majority were ST5 that exhibited specific genetic changes related to host adaptation [42]. Recently, swine associated ST5 MRSA isolates in the USA were also found to differ markedly from ST5 human clinical isolates, suggesting that swine adapted ST5 isolates, like swine adapted ST398, may have reduced virulence for humans [46].

It is evident that multiple lineages of *S*. *aureus* can be endemic in pig populations, and their relative prevalences vary geographically and probably temporally [47]. Given this diversity, the penchant to designate the ST398 lineage of *S*. *aureus* to be uniquely and synonymously ‘livestock associated’ is untenable and should be discouraged. More importantly with respect to elucidating the human health implications of ST398 in livestock, in several countries distinct human-adapted ST398 variants have been shown to circulate in the community, independent of livestock reservoirs, and cause human clinical MSSA infections [48,49]. Consequently, the tendency to casually infer zoonotic origin of human *S*. *aureus* infections based simply on MLST lineage or spa type is imprudent, particularly in the absence of known livestock contact [50]. On the other hand, indirect transmission of *S*. *aureus* of apparent livestock origin has led to human clinical infections in some countries, although the pathways of transmission in such cases are undetermined [51]. Conversely, dismissing the possibility of an animal origin of isolates based solely on MLST or spa type is equally questionable as variants of certain subtypes (e.g., ST5, ST15, and ST72) have been documented in pigs in multiple countries[11]. Essential steps towards more nuanced inference and understanding of the nature of livestock reservoirs of *S*. *aureus* are to widen the focus of research from MRSA towards more holistic studies of *S*. *aureus* populations in animals, more comprehensive genotyping of isolates of animal and human origin, and to advance the understanding of genotypic determinants of host adaptation and virulence in the context of interspecies transmission [48,52,53].

## Supporting Information

S1 Table Spa types and MLST types of MSSA and MRSA isolates from 38 swine farms. (XLSX)
